# Synergistic effect of the next generation insecticide flupyradifurone with a fungal pathogen in the ant *Lasius niger*

**DOI:** 10.1038/s41598-025-20393-z

**Published:** 2025-10-21

**Authors:** Daniel Schläppi, Adam Al-Hashemi, Vaneeza Wasif, Florent Masson, Luke Leckie, Nathalie Stroeymeyt

**Affiliations:** 1 School of Biological Sciences, University of Bristol, Life Sciences Building, 24 Tyndall Avenue, Bristol, BS8 1TQ UK; 2 Present Address: Institute of Bee Health, Vetsuisse Faculty, University of Bern, Schwarzenburgstrasse 161, 3003 Bern, Switzerland; 3 Present Address: Luddy School of Informatics, Cognitive Science, Indiana University Bloomington, Bloomington, 47405 IN USA

**Keywords:** Chronic exposure, Fieldrealistic, *Metarhizium brunneum*, Pesticide-pathogen interaction, Stressor interaction, Sublethal effects, Ecology, Conservation biology, Ecological epidemiology, Environmental impact

## Abstract

Pesticides are important drivers of global insect declines, and their detrimental effects can be amplified by synergistic interactions with other stressors. While there is growing evidence of adverse impacts of the next-generation insecticide flupyradifurone (FPF) on pollinators, its effects on other insects and interactions with other stressors remain poorly understood. We investigated the effects of chronic exposure to a range of FPF concentrations, alone and in combination with the entomopathogenic fungus *Metarhizium brunneum*, in the non-target ant species *Lasius niger*. Twenty-day exposure to FPF concentrations ≥ 100 ppm significantly increased worker mortality, whereas lower concentrations (≤ 50 ppm) had no direct lethal effect. Furthermore, worker survival following acute exposure to *M. brunneum* was altered by prior pesticide exposure: compared to unexposed controls (0 ppm), workers exposed to sublethal FPF concentrations (5 and 50 ppm) for 10 days before fungal challenge were more susceptible to infection, with hazard ratios for fungal-induced mortality rising from 1.69 in controls to more than 3.5 in pesticide-exposed individuals. This demonstrates an indirect sublethal effect of FPF via synergy with the pathogen, raising further concerns about the environmental impact of novel pesticides and their implications for insect conservation.

**Supplementary Information:**

The online version contains supplementary material available at 10.1038/s41598-025-20393-z.

## Introduction

Insects are important indicators for environmental health and provide vital ecosystem services such as pollination, pest control, and nutrient cycling^1,2^. However, global insect biodiversity and abundance are being lost at alarming rates^3,4^. Recent research has identified agrochemicals, such as neonicotinoid insecticides, as an important driver of these declines^5–7^. The detrimental impacts of neonicotinoids on non-target organisms led to restrictions in the use of clothianidin, thiamethoxam, and imidacloprid in the European Union in 2013^8–11^. Following the neonicotinoid ban, new pesticides were introduced to the market, including the butenolide insecticide flupyradifurone (FPF). Although structurally different, neonicotinoids and FPF target the same receptor and both disrupt the central nervous system of insects, making them highly effective against a broad range of pests^12,13^. Upon introduction, FPF was advertised as “bee safe” because of its ecotoxicological profile. However, recent evidence suggests that FPF does pose a risk to pollinators, as exposure to field-realistic concentrations of FPF can lead to detrimental sublethal effects, including impaired cognitive abilities, motion coordination deficits, hyperactivity and apathy, as well as impacts on flight and foraging behaviours^14–18^. Such sublethal impacts frequently elude detection in risk assessments which are typically based on acute toxicity assays^19^.

Proper evaluation of environmental risks is further complicated by complex stressor interactions that remain poorly understood. Traditional pesticides such as neonicotinoids have been reported to interact with co-occurring pesticides, pollutants, pathogens, malnutrition, or climate change, producing outcomes that range from antagonistic to additive and synergistic, the latter being of particular concern as they may amplify environmental impacts by causing a combined effect that is greater than the sum of their individual effects^20–23^. Similarly, there have been reports of interactions between FPF and other pesticides or repellents, ranging from antagonistic to synergistic effects^24–28^. By contrast, only a handful of studies have investigated potential interactions between FPF and disease, and none of these have detected synergistic effects on survival or pathogen load through mechanisms such as immune suppression or resource allocation trade-offs^29–32^. Furthermore, studies examining the direct and indirect impacts of FPF focus almost exclusively on bees^24–26,28–32^, whilst other non-target insects such as ants have been overlooked in ecotoxicological research, in spite of their ubiquity and the essential ecosystem services they provide^33^.

Although specific quantitative field data on pesticide exposure in ants are lacking and the relative importance of direct contact or ingestion remains unknown, exposure routes have been assumed to be similar to those in bees, including contact with spray droplets, contaminated foliage and water, as well as foodborne exposure through various sources such as contaminated prey items, plant tissue, nectar and water^10,34,35^. Additionally, as ground-dwelling insects, ants may experience chronic exposure through contact with contaminated soil^10,34^, suggesting that ant exposure levels are likely comparable to, or potentially exceeding those documented for bees. Unsurprisingly, the few studies that have investigated ants as non-target organisms indicate that traditional pesticides can also negatively impact colony health^10,34^. Yet, they are not routinely included in environmental risk assessments of pesticides and the effects of FPF on ants have not been investigated.

In this study, we focus on the black garden ant, *Lasius niger*, a soil-dwelling species distributed broadly across Europe, commonly nesting in agricultural environments^36^ and frequently used as a model organism in ecological studies^35,37^. Like other soil-dwelling insects, *L. niger* is commonly exposed to a range of pathogens in its natural habitat, including the generalist, obligate-killing entomopathogenic fungus *Metarhizium brunneum*^38^. *M. brunneum* is ubiquitous in natural soil microbial communities^38^ and widely applied as a biological control agent in agricultural systems^39^, increasing the likelihood of its co-occurrence with pesticides such as FPF. To evaluate the consequences of such co-exposure, we (i) quantified the direct effects of FPF on ant survival across a range of concentrations, and (ii) tested for potential indirect, sublethal effects on disease susceptibility, specifically, whether FPF compromises host resistance to pathogens, by exposing the ants to field-realistic FPF concentrations in combination with *M. brunneum*.

## Methods

*L. niger* colonies were initiated using newly mated queens collected in Berlin in July 2021. Colonies were housed in standard nesting tubes with a rear water reservoir and access to a foraging arena and maintained at 25 °C and 65% humidity, with a 12 h day/night cycle, *ad libitum* supply of water and honey water (15% mass fraction of honey) and weekly provision of *Drosophila hydei*^35^. All experiments adhered to the pertinent guidelines and regulations stipulated by local authorities and work with the invertebrate species utilized in this study did not necessitate ethical approval or permits.

### Experiment 1: Flupyradifurone susceptibility test

To gain a comprehensive understanding of the susceptibility of *L. niger* to flupyradifurone, we tested a broad range of concentrations, including levels exceeding field-realistic exposures, using a replicated experimental design. We sampled 120 workers from each of four stock colonies and randomly split them into eight subsets of fifteen workers (*N* = 480 ants across 32 subsets). Each subset was kept in a separate Petri-dish (⌀ = 50 mm) with fluon-coated walls and sealed with parafilm. The floor of each Petri-dish was covered with a thin layer of plaster of Paris to which 1 mL of water was added to maintain humidity. After one day of acclimatisation, each colony’s subsets were pseudo-randomly allocated to one of eight FPF concentration treatments, ranging from 0 (control) to 500 ppm, with a total of 60 ants per treatment group (15 ants from each of 4 colonies). The treatment series included a field-realistic concentration of 5 ppm, which falls within the range detected in nectar consumed by honey bees^40^. Fresh FPF feeding solutions were prepared on each feeding day by mixing different proportions of a 30% honey water solution, a 1000 ppm FPF stock solution and MilliQ water to produce 8 different solutions with final concentrations of 15% honey water each and 0 (control), 0.5, 1, 5, 10, 50, 100 or 500 ppm FPF (Sigma-Aldrich PESTANAL^®^ analytical standard, 99.5% purity). At the start of the experiment and every 3 days thereafter, each subset received a fresh FPF feeding solution provided in a 200 µL Eppendorf tube closed with cotton wool. Survival was checked daily for twenty days by counting and removing dead ants (see timeline - supplementary Fig. 1a). All survival checks were conducted blind to treatment.

### Experiment 2: Flupyradifurone and fungus interaction test

To test for potential interactive effects between FPF and pathogens, ants were chronically exposed to sublethal FPF concentrations for ten days, and then subsequently challenged with *M. brunneum* (strain MA275, KVL 03-143) in a replicated experimental design. *M. brunneum* is frequently used as a model organism in research on ant individual and social immunity, owing to its ecological relevance, well-characterized infection process, and the diverse physiological and behavioural defences that ants exhibit against it^37,38,41–43^. Upon contact, its conidiospores adhere to the insect cuticle and actively penetrate the host withing the first 2 days, causing host death around 3–4 days post infection, followed by sporulation 1–3 days after host death, during which sporulating cadavers may release up to 12 million propagules^44–46^. A preliminary test confirmed that FPF does not interfere with fungal germination (supplementary Fig. 2).

We sampled 132 workers from each of five stock colonies and distributed them evenly across 3 separate Petri-dishes, resulting in a total of 15 Petri-dishes containing 44 ants each (total ants *N* = 660). The three Petri-dishes from each colony were pseudo-randomly assigned to one of three chronic sublethal FPF-exposure treatments (0, 5 and 50 ppm of FPF, which were found not to induce mortality in experiment 1). Feeding solutions were freshly prepared and provided every 3 days as described above. After ten days FPF exposure, 40 workers from each Petri-dish were randomly selected and split into two groups of 20, which were randomly allocated to two pathogen treatments (fungus and sham challenge). This resulted in a full factorial design involving six treatment groups (three FPF treatments $\times$ two pathogen treatments) with 5 replicates each (one per stock colony). Accordingly, the experiment was organised into 5 successive experimental blocks, each involving a single stock colony and one replicate for every treatment for a total of 100 ants per treatment. In each block, all sham- and fungal challenges were performed in a single session lasting less than two hours. The 20 ants of each Petri-dish were exposed consecutively, while the order in which the Petri-dishes for all treatments were exposed was varied pseudo-randomly across all blocks to mitigate temporal effects.

Fungal and sham challenges were performed using standard procedures^37^: *M. brunneum* was cultured on Sabouraud-Dextrose Agar (SDA) plates at 24 °C until sporulation. Conidiospores were harvested in 0.05% Triton X-100, washed twice and adjusted to 10^9^ spores/mL in 0.05% Triton X-100 to create a spore stock suspension. The stock was diluted by half with 0.05% Triton X-100, resulting in a fungal suspension with an estimated spore concentration of 5 × 10^8^, which should be close to the median lethal dose LD50 for this strain, based on experimental survival rates (see supplementary Fig. 3). Ants were placed on ice, then exposed to 0.3 µL of the treatment solution (fungal suspension or sham solution of 0.05% Triton X-100 only) pipetted onto their gaster. They were then left to dry on filter paper for 1 min and subsequently transferred individually into individual Petri-dishes (⌀ = 35 mm, *N* = 600) with fluon-coated walls and a water tube (200 µL Eppendorf tubes closed with cotton wool). These Petri-dishes were then sealed with parafilm and placed in the incubator for two weeks with daily survival checks conducted blind to treatment (see timeline - supplementary Fig. 1b).

### Statistical analyses

All statistical analyses were performed using R v4.2.2^47^. Survival analyses were performed using Mixed-effect Cox proportional hazards models using R packages *survival*^48^ and *coxme*^49^. For the FPF susceptibility test, treatment group was used as a categorical fixed effect and Petri-dish and stock colony as random effects. For the FPF and fungus interaction test, fungal exposure group, FPF concentration and their interaction were used as categorical fixed effects and Petri-dish, stock colony and exposure block as random effects. Fixed effect significance was tested using two-tailed Wald chi-square tests. *Post-hoc* contrasts, defined using package *multcomp*^50^, were used for pairwise comparisons of experimental groups. P-values for post-hoc tests were corrected for multiple comparisons using the Benjamini-Hochberg (BH) method. A significant interaction term between FPF and fungal exposure indicates a deviation from additivity, i.e., that the joint effect of the two stressors cannot be explained by the sum of their independent contributions to hazard. To determine whether this interaction was synergistic or antagonistic, we examined hazard ratios: if the effects of FPF and fungal infection were additive, we would expect the relative mortality risk induced by *M. brunneum* to remain the same across FPF treatments. An increase in *M. brunneum*-induced mortality after exposure to FPF would indicate a synergistic effect, whereas a decrease would indicate an antagonistic interaction.

## Results

### Experiment 1: Flupyradifurone susceptibility test

**Fig. 1:**
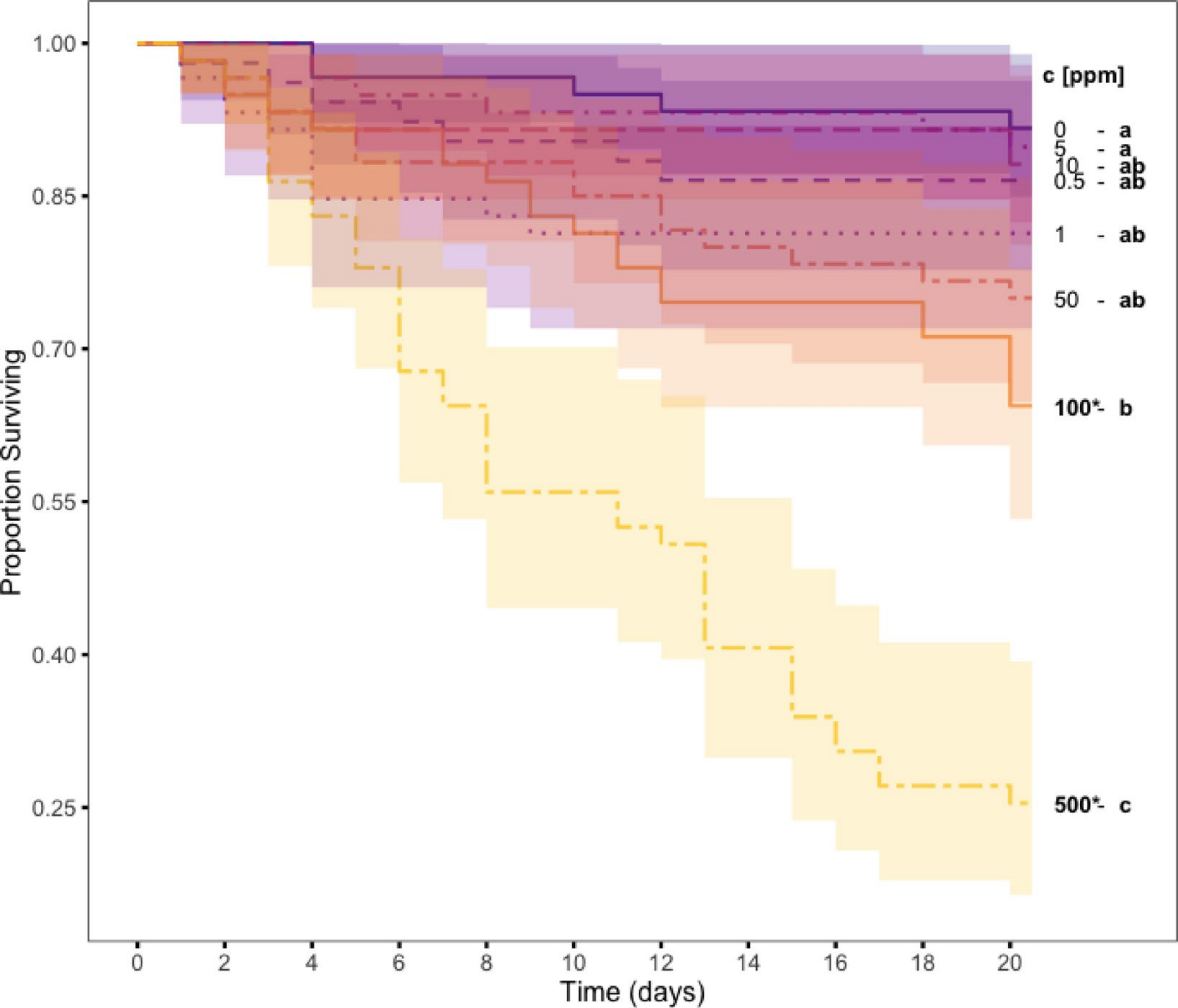
Kaplan-Meier survival curves showing the effect of chronic flupyradifurone (FPF) exposure on *Lasius niger* worker survival. Lines represent the proportion of *Lasius niger* workers surviving as a function of time during chronic exposure to different concentrations of FPF (total sample size: *N* = 467). Shaded areas represent the 95% confidence interval around each line. FPF concentration had a significant overall effect on survival (Cox proportional hazards model; χ² = 31.699, df = 7, *p* < 0.001), with higher concentrations generally leading to increased mortality, although the relationship was not strictly dose-dependent. Results of pairwise comparisons are indicated by letters in the legend (same letter: *p* > 0.05; different letter: *p* ≤ 0.05 in *post hoc* tests with Benjamini-Hochberg correction). Treatments that differ from the controls (0 ppm) are indicated in bold and with an asterisk (*).

FPF concentration had a significant effect on ant mortality (Fig. 1; Mixed-effect Cox proportional hazards model, effect of FPF concentration: χ² = 31.7, df = 7, *p* < 0.001). *Post-hoc* pairwise comparisons revealed that only concentrations of 100 ppm and above led to a significant increase in ant mortality compared to the control (100 ppm vs. 0 ppm: z = 2.81, *p* = 0.017; 500 ppm vs. 0 ppm: z = 4.97, *p* < 0.001). By contrast, concentrations of 50 ppm and below did not increase mortality compared to the control (z < 2.08; *p* > 0.09 in all pairwise comparisons); these were used as sublethal concentrations in experiment 2. The absence of lethal effects in concentrations of 50 ppm and below cannot be attributed to the ants restricting their food intake to limit their exposure to the pesticide, as a supplementary experiment showed that feeding volumes did not differ between ants offered control food (FPF-free) and ants offered food mixed with FPF at 5, 50 or 500 ppm (see supplementary Fig. 4).

### Experiment 2: Flupyradifurone and fungus interaction test

**Fig. 2:**
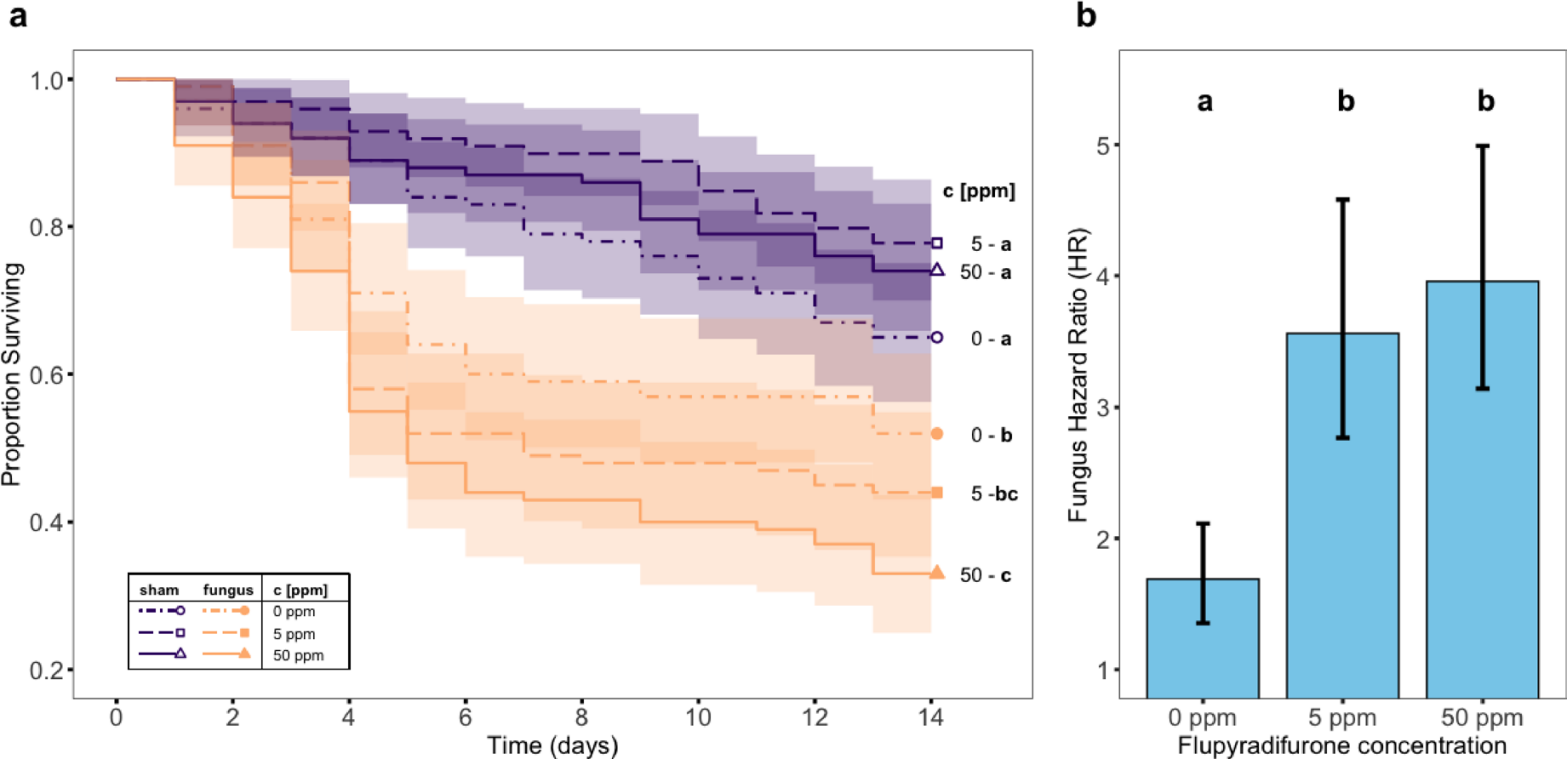
**(a)** Kaplan-Meier survival curves depicting the proportion of *Lasius niger* workers surviving as a function of time following exposure to *Metarhizium brunneum* spores (yellow lines) or to a sham solution (blue lines) after ten days chronic exposure to different concentrations of flupyradifurone (FPF; 0, 5, and 50 ppm; dash dotted, dashed and solid lines, respectively). Shaded areas represent the 95% confidence interval around each curve. **(b)** Fungus Hazard Ratio of fungus-treated ants relative to sham-treated ants in the three FPF exposure conditions. Bars and whiskers represent model estimates and standard errors, respectively. Cox proportional hazards mixed effect model, interaction FPF exposure $\times$ fungal exposure: χ² = 8.22, df = 2, *p* = 0.016. Results of pairwise comparisons are indicated by letters (same letter: *p* > 0.05; different letter: *p* ≤ 0.05; *post hoc* contrast with Benjamini-Hochberg correction).

Our experimental procedure led to higher baseline mortality than in experiment 1 (Fig. 2), with 22–35% mortality in sham-exposed treatments at day 14 compared to 7–20% at similar FPF concentrations in experiment 1. This increase was likely due to stress from sampling, handling and anaesthesia during acute (fungal or sham) challenges, or because ants in Experiment 2, unlike in Experiment 1, were kept in isolation to avoid confounding effects of fungal spore removal by allogrooming. Despite this, exposure to each stressor in isolation had the expected effect on ant survival (Fig. 2a): challenge with the pathogenic fungus *M. brunneum* increased the mortality of control (FPF-free) ants, whilst chronic exposure to sublethal FPF concentrations did not affect the survival of sham-challenged ants (Cox proportional hazards mixed effect model with BH correction for multiple comparisons, fungus- vs. sham-challenged ants, no FPF: Hazard Ratio (HR) = 1.69, z = 2.36, *p* = 0.04; control, low and high FPF, sham-challenged ants: z ≤ |1.97|, *p* ≥ 0.07 in all comparisons). However, there was a significant interaction between FPF treatment and fungal exposure (χ² = 8.29, df = 2, *p* = 0.016). More specifically, we found that *M. brunneum*-induced mortality was significantly greater in FPF-exposed ants than in control ants, showing that the effects of the two stressors were not additive (Fig. 2a-b; FPF 5 ppm: HR = 3.56, z = 5.04, *p* < 0.001; FPF 50 ppm: HR = 3.96, z = 5.94, *p* < 0.001; pairwise comparisons of HRs with BH correction, control vs. FPF 5 ppm: z = 2.22, *p* = 0.048; control vs. FPF 50 ppm: z = 2.65, *p* = 0.024; FPF 5 ppm vs. 50 ppm: z = 0.31, *p* = 0.76). This indicates that infections with *M. brunneum* are more likely to be lethal if the ants have previously been chronically exposed to a sublethal FPF dose, highlighting a synergistic interaction between the two stressors.

## Discussion

This study assessed the direct effect of chronic FPF exposure on the survival of black garden ants (*L. niger*), revealing increased mortality at concentrations of 100 ppm and above. Furthermore, we demonstrated an indirect effect of FPF on ants at lower, sublethal concentrations (≤ 50 ppm), as the challenge with *M. brunneum* was more likely to be lethal following chronic exposure to sublethal FPF concentrations, indicating a synergistic interaction between the two stressors. Overall, the results of this study raise further concerns about the long-term impacts of novel pesticides on the health of insects.

While chronic exposure to FPF concentrations upwards of 100 ppm resulted in increased worker mortality, concentrations of 50 ppm or lower showed comparable mortality rates to the control group and were thus classified as sublethal. Notably, the direct impact of FPF on ants in the field remains unclear as their exposure to FPF in natural environments has not yet been quantified, meaning that field-realistic exposure doses for ants have yet to be determined^10,34^. Residues in pollen and nectar are considered to be the main exposure routes for bees and their reported residue values are as high as 68 ppm for pollen and 21.8 ppm for extrafloral nectaries^40^. Studies involving honey bees typically consider 4 ppm to be a field-realistic concentration, as nectar collected from the gut of returning foragers contained 4.3 ppm of FPF, at which both lethal and sublethal effects have been reported^17,28,40^. Our sublethal concentrations for ants (0.5–50 ppm) cover a comparable range, suggesting that FPF residue values in the field may not increase ant mortality via direct toxicity. Nonetheless, future studies should quantify true field-realistic exposure in ants by quantifying the uptake of active ingredient at both the individual and colony level over the course of a year, based on a combination of all relevant acute and chronic exposure routes to provide a reliable estimate of the risk posed to ants.

At sublethal concentrations, FPF exposure alone did not affect ant survival, but it increased mortality when combined with *M. brunneum* exposure, indicating that FPF may induce an indirect sublethal effect in non-target hosts at field-realistic concentrations via a synergistic interaction with pathogens. More specifically the elevated hazard posed by the fungus in the FPF treatments compared to the control suggests that FPF exposure may compromise host defences, thereby increasing susceptibility to infection. This contrasts with most previous studies in bees, which did not detect FPF-induced changes in survival rates to fungal or viral pathogens^29–32^, and more broadly with findings from studies on other pesticides, where interactions with pathogens have typically been antagonistic rather than synergistic^22,51–53^. However, our findings are consistent with those of one previous study of a traditional neonicotinoid, in which imidacloprid was shown to increase the susceptibility of ants, *Atta sexdens rubropilosa*, to infection by the fungus *Beauveria bassiana*^54^. Similarly, chronic exposure to nicotine has also been shown to enhance the lethal effects of *B. bassiana* in *Cardiocondyla obscurior*^55^, further supporting the idea that exposure to insect neurotoxins can compromise ant immune function and increase their vulnerability to fungal infections.

The synergistic effect between FPF and *M. brunneum* suggests that FPF may affect the behavioural or biochemical immune response of the ants. However, as we did not record the ants’ behaviour or measure their immune function, we cannot identify the underlying mechanism for this interaction. On one hand, FPF could cause a direct immune suppression or trigger a resource allocation trade-off between immune functions and detoxification^56^, as has been suggested for honey bees exposed to neonicotinoids^57,58^. On the other hand, as FPF disrupts the function of the central nervous system, it could also interfere with individual and collective behaviours that mitigate the risk of fungal infection in ants, such as self-grooming, allo-grooming or chemical disinfection^41^. Future studies at the individual and colony levels will be required to determine the underlying mechanisms, to test whether FPF impairs colony-wide social immune responses that protect colonies against epidemics and to assess how individual-level effects impact the colony as a whole^52,59,60^.

Overall, this study contributes to the understanding of stressor interactions by providing evidence for a synergistic effect between FPF and pathogens, and it underscores the ecotoxicological risk posed by novel insecticides. Our findings are concerning as they further challenge the initial perception of FPF’s relative safety and suggest that it may pose risks to beneficial insects that warrant similar scrutiny to that applied to banned neonicotinoids. This stresses the importance of considering sublethal, long-term effects as well as interactive effects between multiple stressors when assessing the risks of pesticides, rather than only evaluating direct mortality. Furthermore, our findings revealed a synergistic interaction between FPF and pathogens in ants, which contrasts with the absence of such an effect in previous studies on honey bees. This shows that the use of bees as surrogates for other non-target organisms is inadequate when evaluating the risk of pesticides and suggests that other economically and ecologically important arthropods such as ants should be included alongside bees as representative model organisms. Moreover, this study underscores that stressor interactions remain insufficiently understood, and that uncovering their underlying mechanisms and pathways will be crucial for predicting ecological outcomes and informing more effective, precautionary conservation strategies.

## Supplementary Information

Below is the link to the electronic supplementary material.

Supplementary Material 1

## Data Availability

The supplementary files, raw data and code supporting the results are archived on figshare and can be accessed via https://figshare.com/s/b04482638fe50545ab49.
