# Supplementary material for: Synergistic effect of the next generation insecticide flupyradifurone with a fungal pathogen in the ant Lasius niger
Source: Sci Rep. 2025 Oct 21;15:36636. doi: 10.1038/s41598-025-20393-z (PMC12541035; doi:10.1038/s41598-025-20393-z)
Supplement: Supplementary file 1 — Supplementary Material 1 [file 41598_2025_20393_MOESM1_ESM.docx]

Supplementary Materials - Synergistic effects of the next generation insecticide flupyradifurone with a fungal pathogen in the ant *Lasius niger*

**Contents**

**1. Experimental Timelines 2**

Supplementary Figure 1: Experimental Timelines

a) Experiment 1: Flupyradifurone susceptibility test assessing the effects of chronic FPF exposure on *Lasius niger* workers

b) Experiment 2: Flupyradifurone and fungus interaction test examining the synergistic effects of FPF and *Metarhizium brunneum* exposure on workers

**2. Antifungal Assay: *Metarhizium brunneum* growth inhibition test 3**

Overview of the disk-diffusion method for testing fungal inhibition by FPF

Supplementary Figure 2: Antifungal assay results

a) Flupyradifurone at 0, 10, 100, and 1000 ppm

b) Sodium hypochlorite (positive control)

**3. *Metarhizium brunneum* lethality test 4**

Testing survival of *L. niger* after exposure to different concentrations of *M. brunneum*

Supplementary Figure 3: Survival of *L. niger* workers after an acute *M. brunneum* challenge

**4. Quantification of Food Uptake 5**

Methodology to assess food uptake in ants exposed to different FPF concentrations

Supplementary Figure 4: Quantification of food uptake by *Lasius niger* workers - Volume (μL) of honey water consumed in 24 h at varying FPF concentrations

**5. References 8**


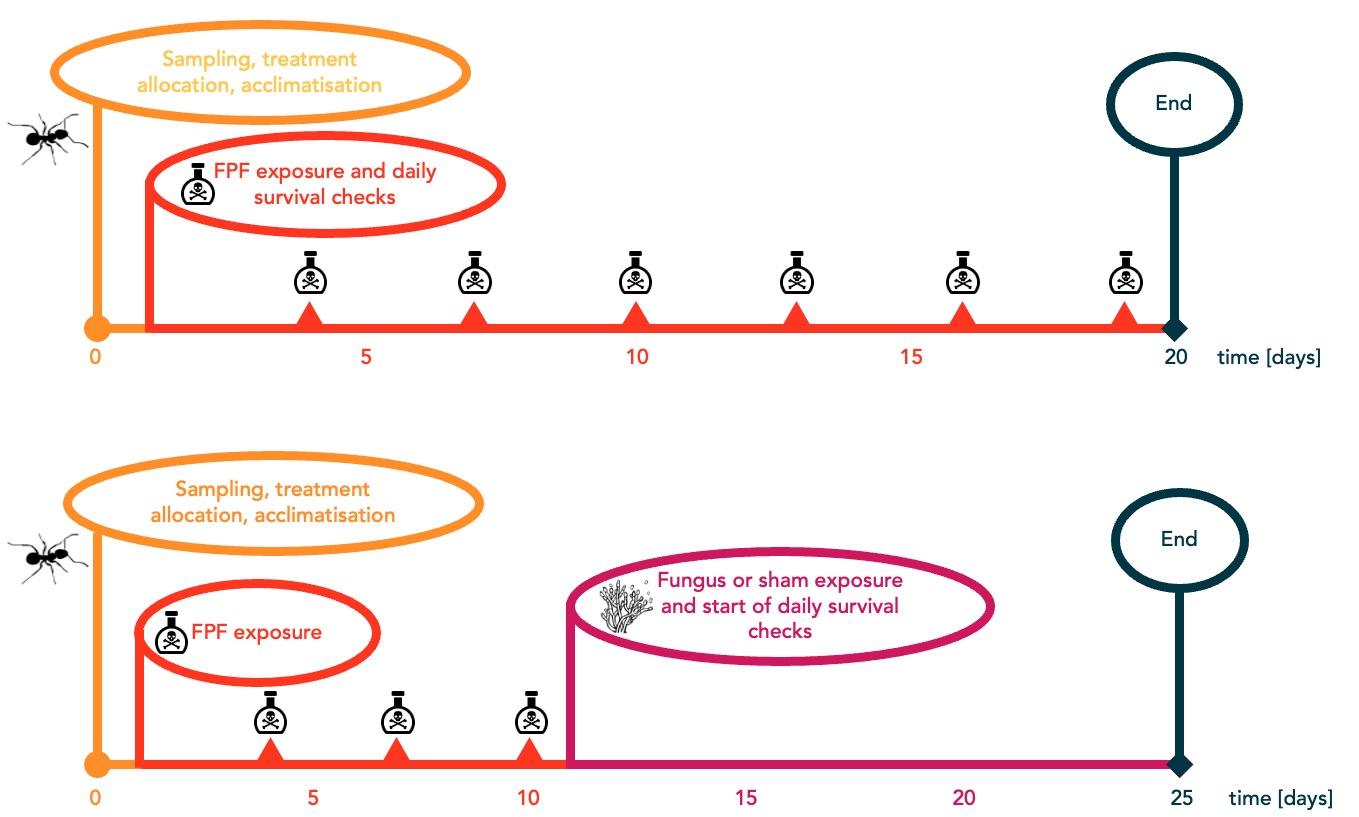


**a**

**b**

Experiment 1: Flupyradifurone susceptibility

Experiment 2: Flupyradifurone and fungus interactions

**b**

**a**

**Supplementary Figure 1.** Experimental timelines illustrating the sequence of key events from the two main experiments. **a)** Experiment 1: Flupyradifurone (FPF) susceptibility test assessing the effects of chronic FPF exposure on the survival of individual *Lasius niger* workers. Day 0: Sampling and allocation of ants into treatment groups. Days 1–20: Chronic exposure via food, with daily survival checks and refreshing of feeding solutions every 3 days. **b)** Experiment 2: FPF and fungus interaction test examining the synergistic effects of FPF and *Metarhizium brunneum* exposure on the survival of individual workers. Day 0: Sampling and allocation of ants into treatment groups. Days 1–10: Chronic exposure to control or pesticide solution, with provision of fresh feeding tubes every 3 days. Day 11: Fungal (or sham) exposure, followed by 14 days of daily survival checks.

# Antifungal assay: *Metarhizium brunneum* growth inhibition test


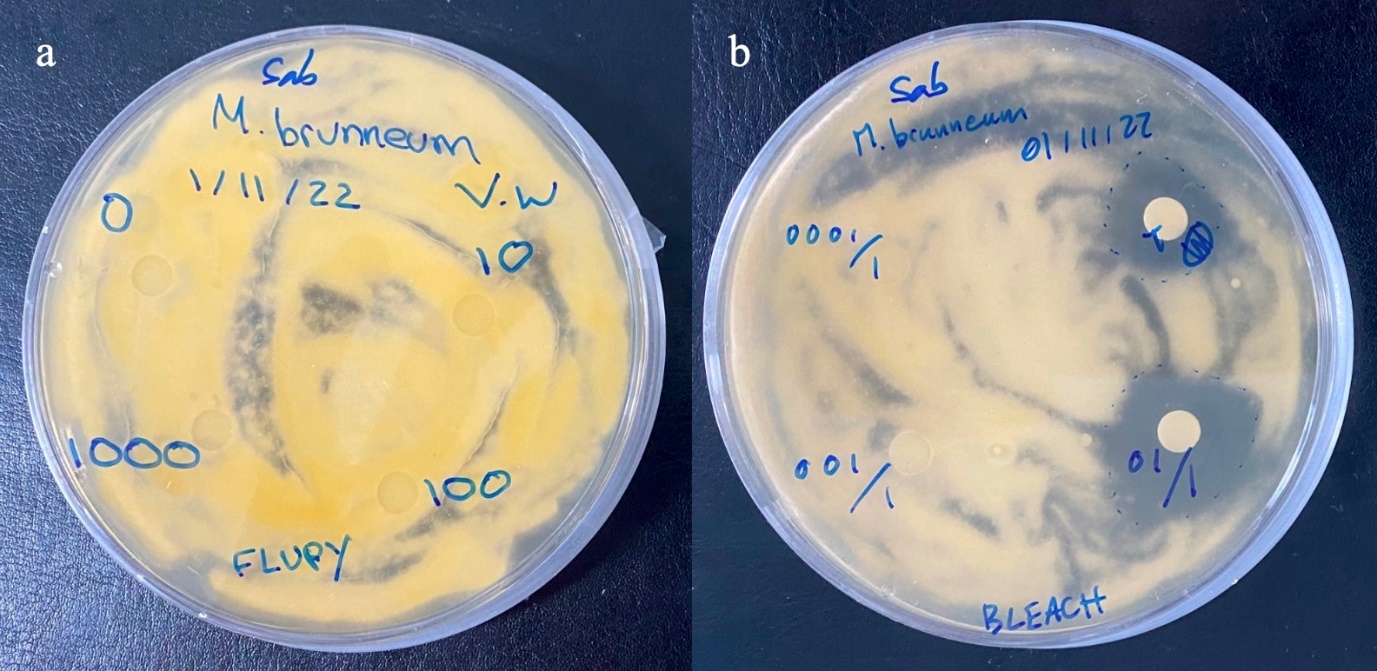
Flupyradifurone (FPF) acts by specifically impairing the nervous system of insects^1^, and thus, no antifungal activity is expected from this agrochemical. Nonetheless, any results of combined exposure treatments would be difficult to interpret or even invalidated if FPF did inhibit the growth of *M. brunneum.* To test the ability of FPF to inhibit *M. brunneum* germination we used an established disk-diffusion assay^2^. Briefly, SDA plates were inoculated with 50 µL of 10^9^/mL *M. brunneum* conidiospore suspension. Then, filter paper disks (6 mm, Cytiva Life Sciences) were soaked with 15 µL of FPF solutions (10, 100, 1000 ppm), distilled water (0 ppm; negative control) or 5% sodium hypochlorite (positive control) and put on the agar using forceps. Plates were sealed, incubated for 48h at 24°C and photographed to measure the growth inhibition diameter. Even at the highest FPF dose no growth inhibition zone was visible, suggesting that *M. brunneum* germinated normally (supplementary Fig. 2). Our findings are in line with findings for neonicotinoids, which have been used successfully in studies with fungal pathogens where no negative effects on conidia germination, conidia production and vegetative growth of Metarhizium fungi were detected^3-5^.

**Supplementary Figure 2. Antifungal assay.** Disk diffusion method to test for germination inhibition of (a) flupyradifurone at 0, 10, 100 and 1000 ppm and (b) sodium hypochlorite (positive control).

# *Metarhizium brunneum* lethality


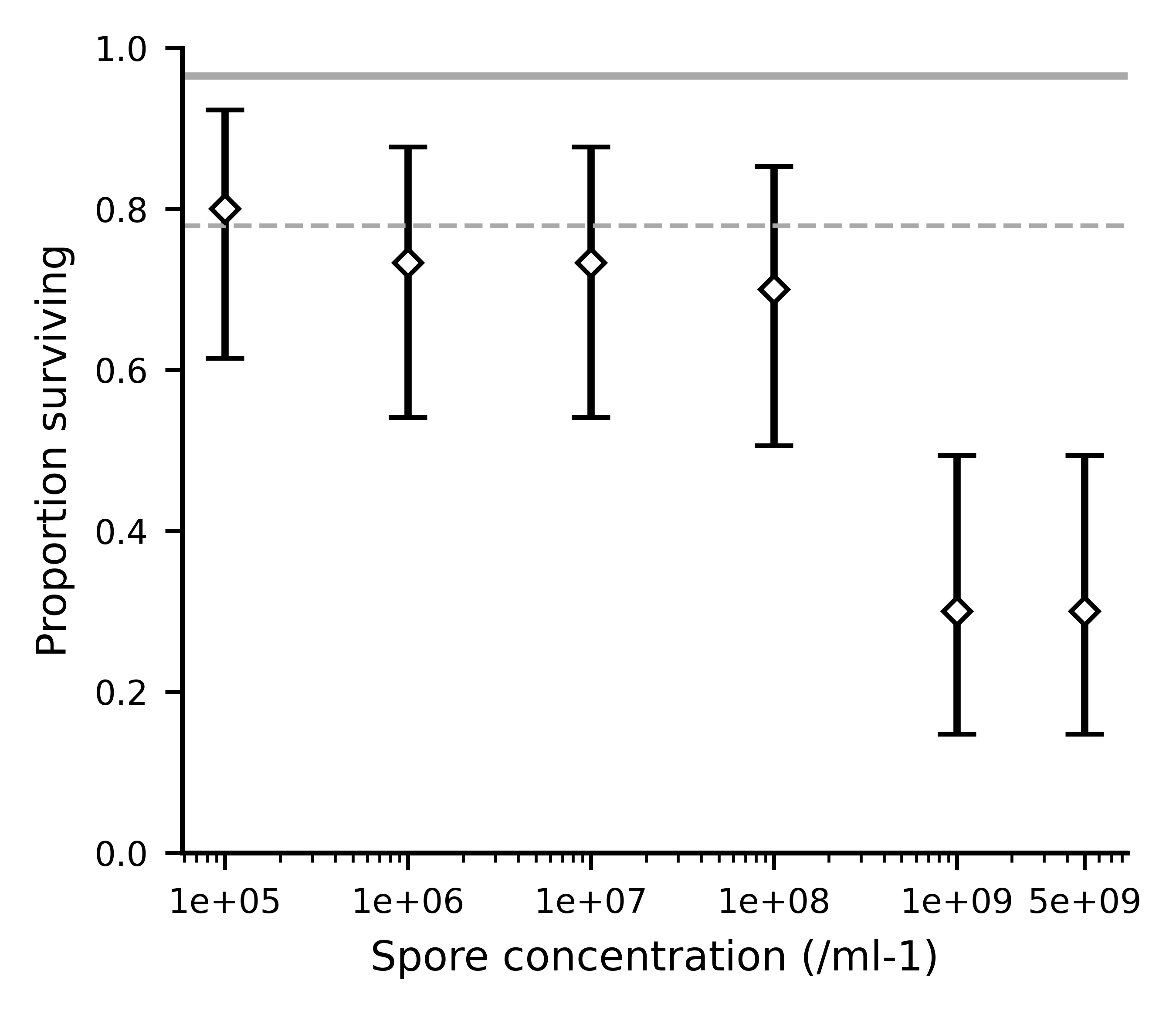
To test the survival of individual *L. niger* at different concentrations of *M. brunneum,* we inoculated individual ants with either a sham solution of 0.05% Triton X-100 or *M. brunneum* in 0.05% Triton X-100*.* Ants may naturally encounter high concentrations of this fungal pathogen as for instance, sporulating cadavers can release up to 12 million infectious spores^6^, hence, we tested the following range of concentrations: 1x10^5^,^,^ 1x10^6^, 1x10^7^, 1x10^8,^, 1x10^9^, or 5x10^9^ spores/mL. For each of the six treatment groups a total of 30 ants were inoculated using the following protocol: Ants were first chilled on ice to reduce their mobility. Using sterile forceps, individuals were then picked up by their legs and 0.3 μL of the treatment solution was pipetted onto their abdomen. Inoculated ants were briefly placed on sterile filter paper to remove excess solvent and then transferred to a separate 9 cm diameter Petri-dish with ad libitum access to 10% sugar water and water. Ants were maintained individually in their Petri-dishes for a duration of 12 days, after which the experiment was terminated. At the conclusion of the 12-day period, the survival rate of ants was calculated for each treatment group, based on the proportion of surviving ants (supplementary Fig. 3). Given the survival of ants for this strain and exposure protocol, the LD50 should be somewhere between 1x10⁹ and 1x10⁸ spores/mL. Hence, we used 5x10⁸ spores/mL for our FPF and fungus interaction experiments.

**Supplementary Figure 3. Survival of *L. niger* workers after an acute *M. brunneum* challenge.** Dose–response showing the mean survival of *L. niger* workers 12 days after exposure to a sham solution of 0.05 % Triton X-100 (grey solid line) or to serial spore dilutions of *M. brunneum* ranging from 1x10^5^ to 5x10^9^ spores/mL (white diamonds). Whiskers (dashed grey line for sham) indicate 95% confidence intervals.

# Quantification of food uptake

## Methods

Pesticide contamination can modulate feeding motivation and food uptake, as shown for honey bees^7-9^. Thus, to determine whether disparities in survival may be due to differences in food consumption, we quantified honey water uptake over a day at different FPF concentrations. For this purpose, we quantified fluorescence of homogenized ants that had access to a feeding solution containing fluorescein dye^10^. The feeding solutions were prepared as described in the main manuscript, but with a fraction of water replaced by fluorescein sodium salt (Sigma-Aldrich) stock solution at 2% in MilliQ water. Four treatment solutions with final concentrations of 15% honey water, 0.01% fluorescein and varying concentrations of FPF (0, 5, 50 or 500 ppm, that is control, low, mid, and high treatment) were prepared. Workers were sampled from 5 source colonies and split into 4 subsets per colony in a stratified random way. Subsets were kept in Petri-dishes in the incubator for three days without food. Subsequently, each subset was assigned pseudo-randomly one of the four treatments groups. Final sample size was 106, 108, 105 and 102 workers in the control, low, mid and high treatment respectively. The slight variation in sample sizes across treatments was due to a small number of worker deaths during the pre-experiment starvation period. Workers had access to the solution for 24 h before being freeze killed at -80°C for 30 min. Samples were then homogenized individually in racked collection tubes (Qiagen) containing 100 μL of phosphate-buffered saline (PBS) buffer and a 2 mm glass bead each. Homogenization was performed using a tissue lyser at 30 Hz for two runs of 1 minute with a plate rotation between the two runs. Following 2 minutes of centrifugation at 3000 rpm, 20 μL of the supernatant and 80 μL PBS buffer were transferred into 96-well plates. Fluorescence was measured on a SpectraMax iD5 plate reader with excitation at 485 nm and emission at 535 nm. Each measure was averaged from 4 reading points 1.5 mm apart, with 400 ms integration time and a gain of 500 V.

Quantification was done using a standard curve, which was obtained using an aliquot of the control treatment feeding solution containing 0.01% fluorescein and 0 FPF, which was treated identically to the meals that were served to the ants (same light and temperature conditions throughout the duration of the experiment, and subsequent freezing). Reference standard curves were prepared from the retained aliquot by making a serial dilution for 8 solutions containing 5, 2.5, 1.25, 0.625, 0.3125, 0.15625, 0.078125, or 0 μL of 0.01% fluorescein treatment solution mixed with PBS to a final total volume of 100 μL. An unfed ant treated identically to the samples was used as a blank. To calculate the meal volumes, mean fluorescence value of blanks was subtracted from all other samples and consumed volume was extrapolated from the standard curves.

Statistical analyses were performed in R v4.2.2^11^. A generalized linear mixed model (GLMM) with a negative binomial distribution was used to test for differences in meal size depending on FPF concentration. Treatment was included as fixed effect and Petri-dishes, colonies as well a block as random effects. Post hoc testing p-values were adjusted using the Benjamini-Hochberg method to correct for multiple comparisons.

## Results


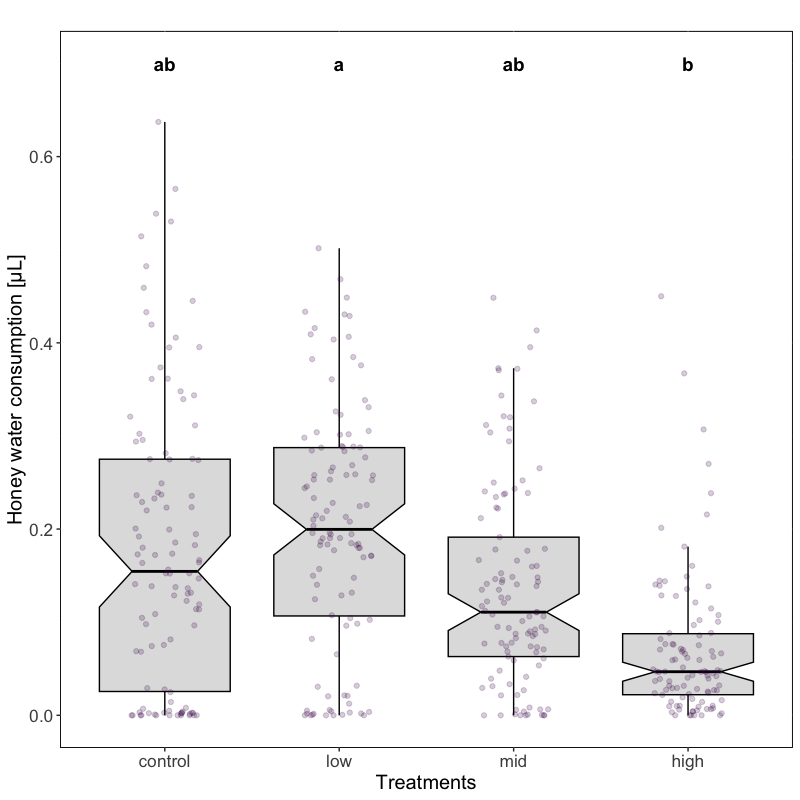
The estimated consumed volume of honey water over 24 hours, was significantly different among treatments (χ² = 7.853, df = 3, p = 0.049; supplementary Fig. 4). Pairwise comparisons using Tukey adjustment indicated that ants in the treatment with the highest FPF concentration (500 ppm) consumed lower volumes (0.06 ± 0.07 μL) compared to ants in the low treatment (0.17 ± 0.14 μL; p = 0.048). The remaining pairwise comparisons with the control and the mid treatment (0.159 ± 0.14  μL and 0.15 ± 0.14 μL respectively) were not significant.

**Supplementary Figure 4. Quantification of food uptake.** Volume (μL) of honey water consumed by *Lasius niger* workers in 24 h at different concentrations of FPF (control = 0, low = 5, mid = 50, high = 500 ppm). Boxplots are shown with the interquartile-ranges (box), medians (black line in box) and outliers (dots). Transparent dots represent individual data points and bold letters (a,b) indicate significant differences (*p* < 0.05) between treatments (Tukey post hoc test).

In contrast to honey bees, which respond to field-realistic concentrations of FPF by a decreased food intake and erratic foraging behaviour^9,12^, we found that ants do not alter their food uptake if FPF is present in food. Even at 500 ppm the food uptake was comparable to the controls. Consequently, our results suggest that ants are not repelled by sublethal concentrations of FPF, i.e. below 50 ppm. The absence of a repellent effect implies that the ants are prone to collect contaminated food from the environment and sharing it among nestmates, with potentially severe consequences for the colony. The insecticide is likely to reach all members of the colony, including the queen. Even though the queen might have some protection via the colony or potentially superior detoxification compared to workers^13^, she will still get exposed repeatedly over extended periods and thus might face a trade-off between detoxification and reproduction^14^.

# References

1. Nauen, R. *et al.* Flupyradifurone: a brief profile of a new butenolide insecticide. *Pest Manag. Sci.* **71**, 850–862 (2015).

2. Balouiri, M., Sadiki, M. & Ibnsouda, S. K. Methods for in vitro evaluating antimicrobial activity: A review. *J. Pharm. Anal.* **6**, 71–79 (2016).

3. Cramer, L. The synergistic effect of pesticides on the fitness of the ant species *Cardiocondyla obscurior*. *Institut für Biologie, Naturwissenschaftliche Fakultät* vol. Magister (Karl-Franzens-Universität Graz, Graz, 2020).

4. Neves, P. M., Hirose, E., Tchujo, P. T. & Moino JR, A. Compatibility of entomopathogenic fungi with neonicotinoid insecticides. *Neotrop. Entomol.* **30**, 263–268 (2001).

5. Santos, A. V., de Oliveira, B. L. & Samuels, R. I. Selection of entomopathogenic fungi for use in combination with sub-lethal doses of imidacloprid: perspectives for the control of the leaf-cutting ant *Atta sexdens rubropilosa* Forel (Hymenoptera: Formicidae). *Mycopathologia* **163**, 233–240 (2007).

6. Hughes, W. O., Eilenberg, J. & Boomsma, J. J. Trade-offs in group living: transmission and disease resistance in leaf-cutting ants. *Proc. R. Soc. Lond. B Biol. Sci.* **269**, 1811–1819 (2002).

7. Kessler, S. C. *et al.* Bees prefer foods containing neonicotinoid pesticides. *Nature* **521**, 74–76 (2015).

8. Siviter, H. & Muth, F. Exposure to the novel insecticide flupyradifurone impairs bumblebee feeding motivation, learning, and memory retention. *Environ. Pollut.* **307**, 119575 (2022).

9. Wu, Y.-Y., Pasberg, P., Diao, Q.-Y. & Nieh, J. C. Flupyradifurone reduces nectar consumption and foraging but does not alter honey bee recruitment dancing. *Ecotoxicol. Environ. Saf.* **207**, 111268 (2021).

10. Jové, V., Venkataraman, K., Gabel, T. M. & Duvall, L. B. Feeding and quantifying animal-derived blood and artificial meals in *Aedes aegypti* mosquitoes. *J. Vis. Exp. JoVE* e61835 (2020) doi:10.3791/61835.

11. R Core Team. *R: A language and environment for statistical computing*.Version 4.2.2, R Foundation for Statistical Computing, Vienna, Austria (2020). https://www.R-project.org/

12. Hesselbach, H., Seeger, J., Schilcher, F., Ankenbrand, M. & Scheiner, R. Chronic exposure to the pesticide flupyradifurone can lead to premature onset of foraging in honeybees *Apis mellifera*. *J. Appl. Ecol.* **57**, 609–618 (2020).

13. Schläppi, D., Kettler, N., Straub, L., Glauser, G. & Neumann, P. Long-term effects of neonicotinoid insecticides on ants. *Commun. Biol.* **3**, 335 (2020).

14. Schwenke, R. A., Lazzaro, B. P. & Wolfner, M. F. Reproduction–immunity trade-offs in insects. *Annu. Rev. Entomol.* **61**, 239–256 (2016).
